# Expression of *Arabidopsis* Hexokinase in Tobacco Guard Cells Increases Water-Use Efficiency and Confers Tolerance to Drought and Salt Stress

**DOI:** 10.3390/plants8120613

**Published:** 2019-12-16

**Authors:** Nitsan Lugassi, Brijesh Singh Yadav, Aiman Egbaria, Dalia Wolf, Gilor Kelly, Efrat Neuhaus, Eran Raveh, Nir Carmi, David Granot

**Affiliations:** 1 Institute of Plant Sciences, Agricultural Research Organization, The Volcani Center, Bet Dagan 50250, Israel; lugassin@gmail.com (N.L.); brijeshbioinfo@gmail.com (B.S.Y.); aiman@volcani.agri.gov.il (A.E.); daliaw@volcani.agri.gov.il (D.W.); gilor@volcani.agri.gov.il (G.K.); vhncarmi@volcani.agri.gov.il (N.C.); 2 Department of Fruit Tree Sciences, Institute of Plant Sciences, Agricultural Research Organization, Gilat Research Center, Negev, Israel; efratn@volcani.agri.gov.il (E.N.); eran@volcani.agri.gov.il (E.R.)

**Keywords:** guard cell, hexokinase, tobacco, abiotic stress, drought, salt, transpiration

## Abstract

Abiotic stresses such as drought and saline water impose major limitations on plant growth. Modulation of stomatal behavior may help plants cope with such stresses by reducing both water loss and salt uptake. Hexokinase (HXK) is a sugar-phosphorylating enzyme involved in guard cells’ sugar-sensing, mediating stomatal closure and coordinating photosynthesis with transpiration. We generated transgenic tobacco lines expressing the Arabidopsis hexokinase1 (*AtHXK1*) under the guard cell-specific promoter *KST1* and examined those plants using growth room and greenhouse experiments. The expression of *AtHXK1* in tobacco guard cells reduced stomatal conductance and transpiration by about 25% with no negative effects on photosynthesis or growth, leading to increased water-use efficiency. In addition, these plants exhibited tolerance to drought and salt stress due to their lower transpiration rate, indicating that improved stomatal function has the potential to improve plant performance under stress conditions.

## 1. Introduction

Water is frequently a limiting resource for agriculture, and abiotic stresses such as drought and salinity are commonly faced by plants during their life cycles, with significant deleterious effects on growth and productivity [1]. In many plant species, most of the water taken up by the roots (>98%) is lost to the atmosphere via the stomata [2] and many efforts have been made to improve stomatal behavior to reduce water loss [3,4,5,6,7]. Reducing water loss may not only save water and help plants cope with drought, but also reduce salt uptake and confer tolerance to salinity.

Stomata are adjustable pores comprised of two guard cells on the surface of plant leaves that allow gas exchange between the leaf and the atmosphere. Light stimulates stomatal opening, which is followed by the movement of atmospheric carbon dioxide (CO_2_) needed for photosynthesis into the leaf. Mechanistically, stomata open in response to increases in the osmolarity of the guard cells. [8]. For a long while it was thought that sugars are osmolytes that open stomata [9], but recent studies have demonstrated that sugars stimulate stomatal closure mediated by hexokinase (HXK) [10,11,12,13]. HXK is an essential enzyme that phosphorylates glucose and fructose, the products of sucrose cleavage [14]. In plants, HXK is the only enzyme that can phosphorylate glucose and may also phosphorylate fructose [15,16]. During the day, at times when the rate of photosynthesis is high, sugar levels might exceed the plant’s phloem-loading and translocation capacity and sucrose might accumulate in the leaves [17,18,19]. Some of the sucrose is carried by the transpiration stream toward the guard cells [17,18,19,20], enters the guard cells via a sucrose transporter and is cleaved within the guard cells, or is cleaved by apoplastic invertase to glucose and fructose, which may then enter the guard cells via hexose transporters. The hexose monomers (glucose and fructose) are substrates of HXK and as such can be sensed by HXK within guard cells and stimulate stomatal closure forming a feedback mechanism that coordinates sugar production with transpiration [10,11,12,21].

Most studies of HXK in plants have involved *Arabidopsis thaliana* HXK1 (*AtHXK1*), which mediates sugar-sensing, in addition to possessing catalytic hexose-phosphorylation activity [22,23]. Increasing the expression of *AtHXK1* specifically in the guard cells of tomato (*Solanum lycopersicum*), Arabidopsis and citrus [Troyer citrange (*C. sinensis* x *Poncirus trifoliata*)], under the potato-derived *KST1* guard cell-specific promoter, stimulates stomatal closure and reduces stomatal conductance and transpiration. It has been shown that sugars stimulate a guard cell-specific response that is mediated by HXK and ABA and leads to stomatal closure [10,24]. In the current study, we expressed *AtHXK1* specifically in the guard cells of tobacco (*Nicotiana tabacum*) plants and examined its effects on stomatal conductance, transpiration, water-use efficiency (WUE), and tolerance of drought and salt stress.

## 2. Results

### 2.1. Expression of AtHXK1 in Tobacco Guard Cells Reduces Stomatal Conductance and Transpiration and Increases Intrinsic WUE

**Figure 1:**
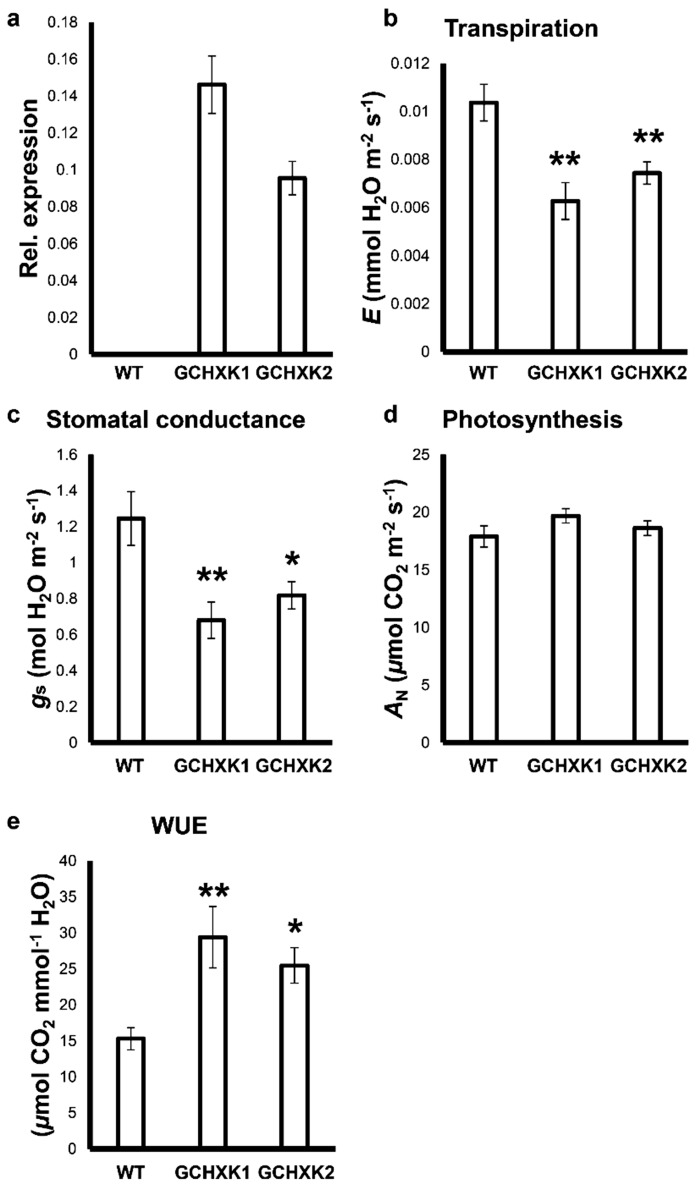
Transgene expression and gas-exchange data from GCHXK tobacco plants. (**a**) Relative expression of *AtHXK1* was examined using RNA extracted from mature leaves of wild-type (WT) and GCHXK plants (GCHXK1 and GCHXK2). Actin (XM_016658252) was used for normalization. For the gas-exchange experiment, 1.5-month-old plants from the different lines were examined. (**b**) Transpiration, (**c**) stomatal conductance and (**d**) photosynthesis of WT and GCHXK lines. (**e**) Intrinsic water-use efficiency (WUEi) was calculated by dividing *A*_N_ by *g*_s_. Data points are means (*n* = 11 for a; *n* = 8 for b–e) ± SE. Asterisks denote significant differences relative to the WT. One asterisk: *t*-test, *p* < 0.05. Two asterisks: *t*-test, *p* < 0.01.

The *KST1* partial promoter (*KSTpro*) has been previously shown to drive guard cell-specific expression in tobacco plants containing a *KSTpro::GFP* construct [25]. We generated independent transgenic tobacco lines that express the Arabidopsis HXK1 (*AtHXK1*) under the *KST1* partial promoter (Figure S1). These lines were referred to as GCHXK lines, with GC standing for guard cells. The expression of *AtHXK1* was verified in the independent homozygous lines GCHXK1 and GCHXK2 using quantitative real-time PCR (Figure 1a).

Gas-exchange analysis of plants grown in the greenhouse revealed that the photosynthesis (*A*_N_) of the GCHXK plants remained unaffected, while the stomatal conductance (*g*_s_) and transpiration (*E*) of both lines were reduced by about 25% (Figure 1b–d), leading to increased intrinsic water-use efficiency (WUE; Figure 1e), calculated as *A*_N_/*g*_s_ [26].

### 2.2. Lysimeter Analyses of GCHXK Plants under Regular Irrigation and Drought Conditions

**Figure 2:**
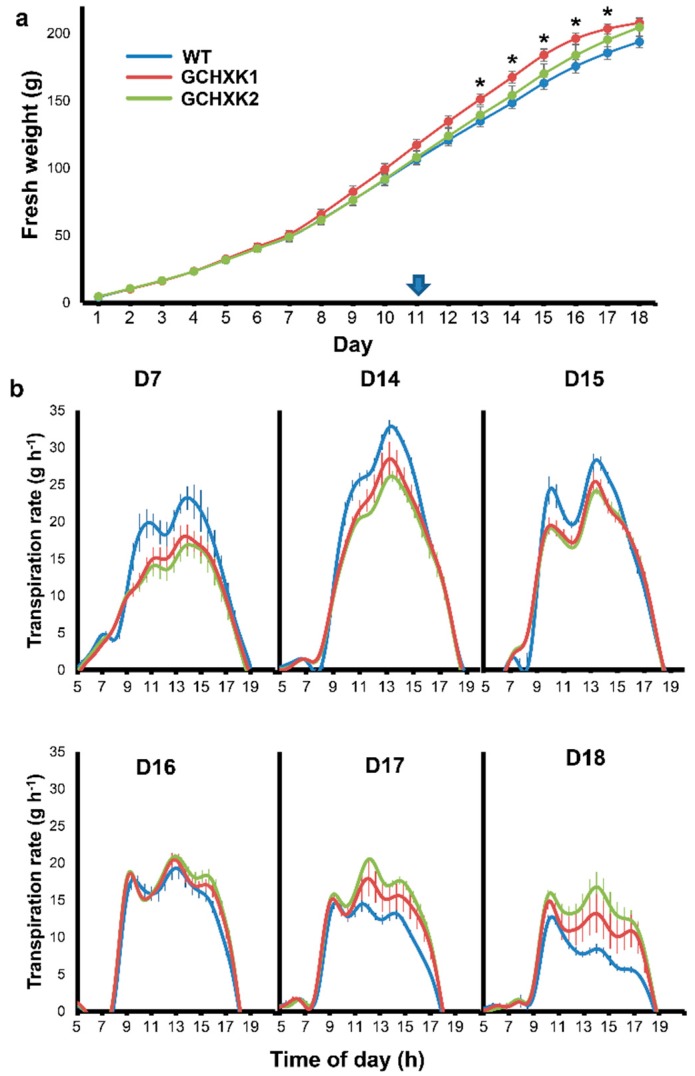
GCHXK plants under regular irrigation and increasingly severe drought conditions. WT (solid line, blue), GCHXK1 (solid line, red) and GCHXK2 (solid line, green) plants were exposed to increasing drought conditions for eight days, after the irrigation was turned off on Day 11 (arrow). The experiment ended on Day 18. Plants were monitored continuously throughout the day using a lysimeter system. (**a**) Fresh weight of the entire plant throughout the lysimeter experiment. (**b**) Transpiration rate during the day on Day 7 (prior to drought) and Days 14–18 (four days after watering stopped to 8 days after watering stopped). Data displayed as means ± SE, *n* = 5. Asterisks denote significant differences relative to the WT (*t*-test, *p* < 0.05).

To examine the whole-plant diurnal behavior of GCHXK1&2 plants under normal irrigation and under drought conditions, whole-plant weight gain and the diurnal transpiration of each individual plant were monitored continuously using a precise and sensitive lysimeter-scale system located in a greenhouse [27]. The fresh weights of GCHXK1, GCHXK2, and WT plants were similar at the start of the experiment and increased at a similar rate over the course of the experiment (Figure 2a), with a slight but significant increase in the weight of GCHXK1 plants at advanced stages of the experiment.

Despite their similar weights, the diurnal transpiration of the two GCHXK lines under normal irrigation conditions was less than that of the WT plants, primarily in the middle of the day, as exemplified on Day 7 (D7) in Figure 2b. Irrigation was stopped on Day 11, yet the transpiration of the WT plants remained higher than that of the GCHXK plants for four more days, until Day 15 (Figure 2b). The transpiration of WT plants was similar to that of the GCHXK plants on Day 16 and fell below that of the GCHXK plants on Days 17 and 18.

**Figure 3:**
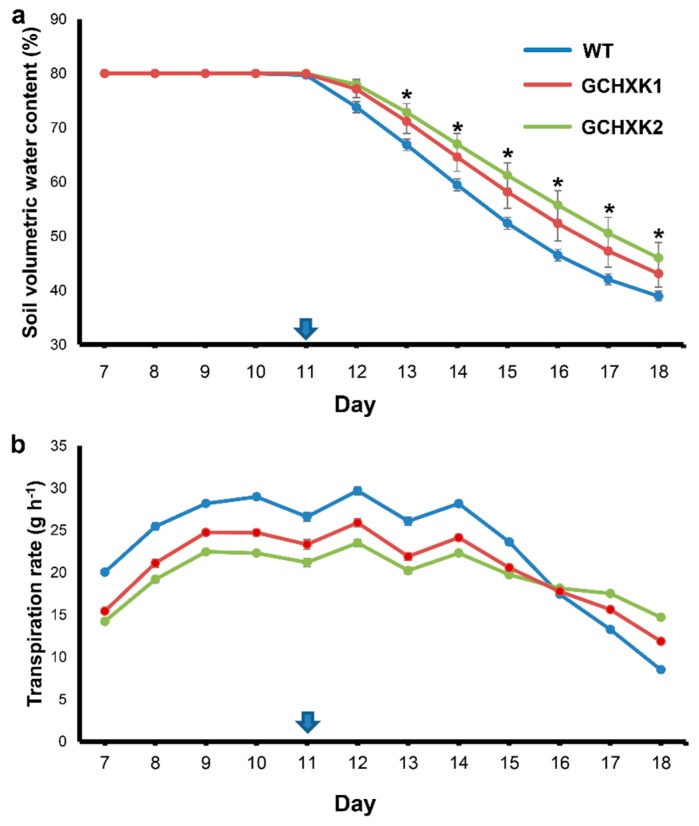
GCHXK plants shows reduced water consumption under drought condition. WT (solid line, blue), GCHXK1 (solid line, red) and GCHXK2 (solid line, green) plants were exposed to increasing drought conditions for 8 days, after the irrigation was turned off. The irrigation was shut off on Day 11(arrow) and the experiment ended on Day 18. Plants were monitored continuously throughout the day using a lysimeter system. (**a**) Soil volumetric water content on Days 9–18. Soil water content was maintained at 80% until Day 11, when the irrigation was shut off. (**b**) Daily average transpiration rate at midday, measured between 10:00 and 14:00 each day for 12 days (Day 7 through Day 18). The drought period started on Day 11. Data given as means (± SE), (*n* = 5). Asterisks denote significant differences relative to the WT (*t*-test, *p* < 0.05).

Due to the initial lower transpiration rate of GCHXK, it was anticipated that the soil of WT plants would dry out more quickly than that of GCHXK plants once the irrigation was turned off. Therefore, we measured the soil water content (SWC) of each pot throughout the experiment (Figure 3a). SWC prior to the cessation of irrigation on Day 11 was high, as expected in light of the constant irrigation. SWC started to decline following the cessation of irrigation, but the decline in the SWC of the pots containing GCHXK plants proceeded more slowly over the entire drought period, indicating that the GCHXK plants consumed less water than the WT plants and began to experience water stress 2–3 days later than the WT plants. No clear effects of the declining SWC on the transpiration rates of WT and GCHXK lines were observed until Day 14, as is shown by the daily mean transpiration rate between 10:00 and 14:00 of the GCHXK and WT plants (Figure 3b). However, from Day 15 onward, the transpiration rates of both the WT and GCHXK lines started to decline, with that of the WT plants declining more quickly than those of the GCHXK plants (Figure 3b). Accordingly, the higher transpiration rate of GCHXK towards the end of the drought period, on Days 17 and 18 (Figure 2), was most likely due to the higher SWC of the pots of GCHXK plants. The similar weight gain of GCHXK2 and the higher weight gain of GCHXK1 over the course of the experiment (Figure 2a) despite lower transpiration rates further supports the notion of enhanced WUE of GCHXK plants, and also indicates that maximal stomatal conductance is not absolutely necessary for high weight gain.

### 2.3. GCHXK Lines Exhibit Salt Tolerance

**Figure 4:**
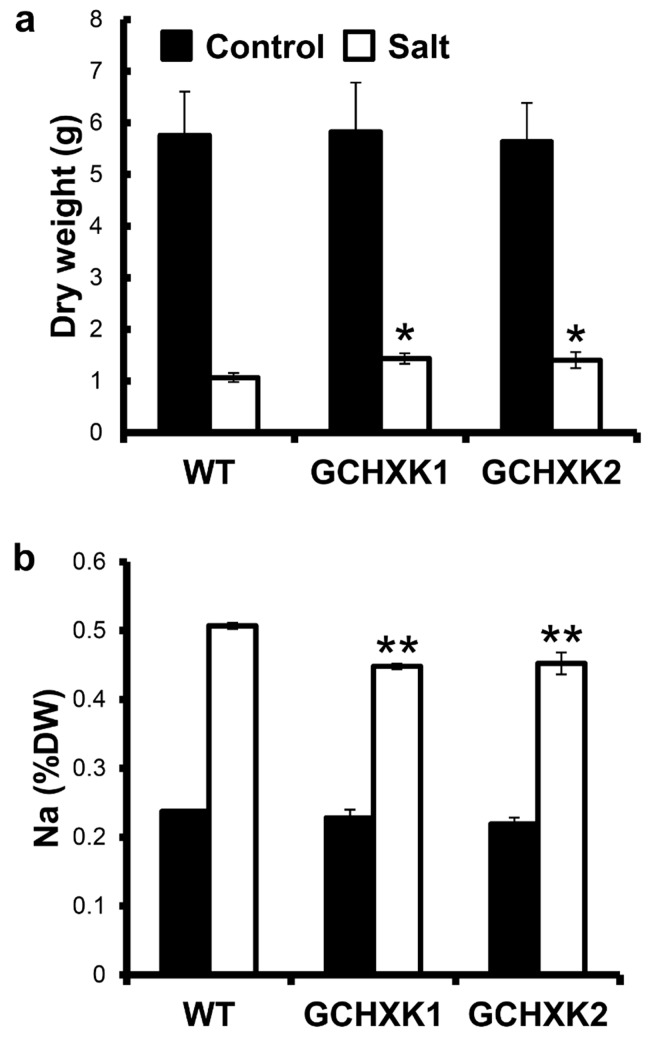
GCHXK plants under salt stress in the growth chamber. Three-week-old WT and GCHXK plants grown in a growth chamber were watered with 200 mM NaCl. We monitored their growth and Na contents for 1 month. (**a**) Dry weights of WT, GCHXK1 and GCHXK2 plants (*n* ≥ 8) at the end of the experiment, under control (black) and saline (white) conditions. (**b**) Sodium concentrations in the shoots of WT, GCHXK1 and GCHXK2 plants at the end of the experiment, under control (black) and saline (white) conditions. Four samples were taken for each line; each sample was composed of 3–4 shoots. Data given as means (±SE). Asterisks denote significant differences relative to the WT (*t*-test, * *p* < 0.05; ** *p* < 0.01).

Knowing that GCHXK plants consume less water due to lower transpiration rates, we assumed that the transgene may confer tolerance to salty water due to lower uptake of salt. To examine this possibility, we irrigated three-week-old WT and GCHXK plants grown in a growth chamber, which allowed for close control of the environmental conditions, with 200 mM NaCl and monitored their growth and Na content. When irrigated with regular water, the dry weights of the GCHXK plants were similar to those of the WT plants (Figure 4a). Irrigation with salty water inhibited growth. The weights of the plants irrigated with the salty water were approximately one-fifth of the weights of the control plants (Figure 4a). Yet, GCHXK1 and GCHXK2 plants gained significantly more weight under the saline conditions than the WT plants did, indicating the partial tolerance of the transgenic lines to NaCl (Figure 4a). To examine the hypothesis that GCHXK plants take in less salt, the Na contents of the WT and GCHXK plants were analyzed. WT and GCHXK plants irrigated with salty water had twice as much Na in their shoots than plants irrigated with water that did not contain any salt (Figure 4b). Yet, the GCHXK plants accumulated significantly less Na than the WT plants (Figure 4b), suggesting that, in fact, less salt was taken up by the transgenic plants.

### 2.4. Greenhouse Experiment with Salty Water

The growth room results regarding irrigation with salty water encouraged us to explore the behavior of GCHXK plants under greenhouse conditions over a longer period of time, which would better represent natural growth conditions. GCHXK plants were planted in perlite (an inert growth substance) and were allowed to become established and grow for 40 days, at which point we started irrigating half of the plants with 150 mM NaCl, knowing that transpiration in the greenhouse is higher than that in the growth room, and therefore 200 mM NaCl might be too toxic. We continued to water the other half of the plants with regular tap water.

**Figure 5:**
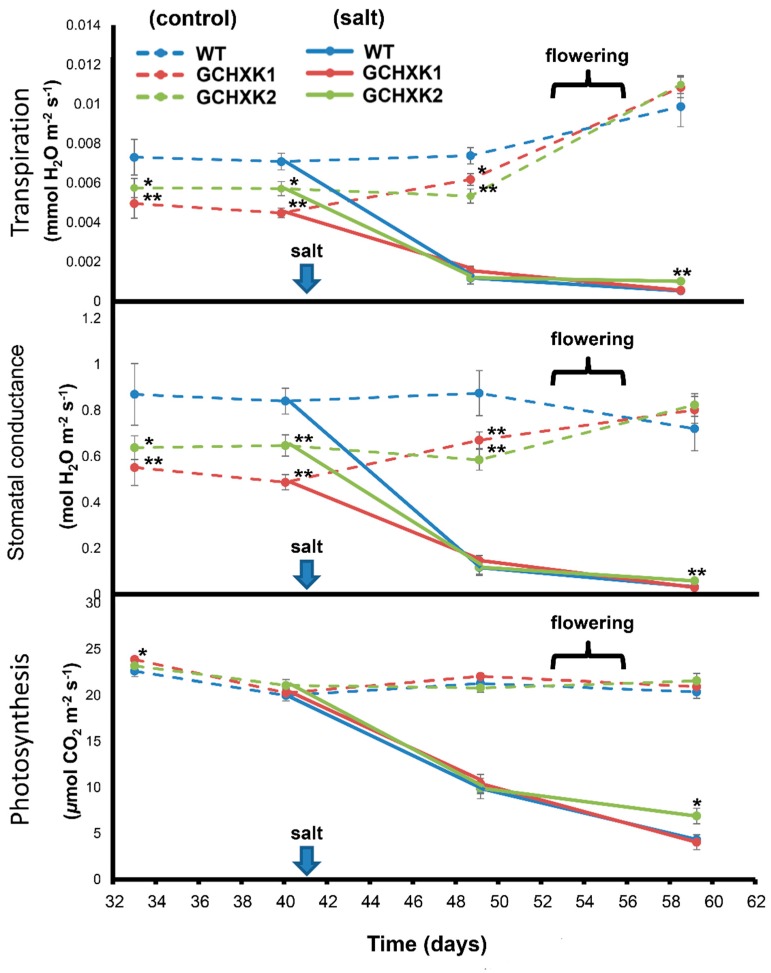
Gas-exchange parameters of GCHXK plants under salt stress. GCHXK and WT plants were planted in perlite (an inert growth substance) and allowed to become established and grow for 41 days. At the end of that period of time, we started irrigating half of the plants with 150 mM NaCl and continued to irrigate the other half of the plants with regular water. During this experiment, we measured transpiration, stomatal conductance and photosynthesis. Data given as means (± SE), *n* ≥ 8. Asterisks denote significant differences relative to the WT (*t*-test, * *p* < 0.05; ** *p* < 0.01).

Gas-exchange analysis using the LI-COR6800 system was carried out at a few points in time during the growth period and the transpiration rate, stomatal conductance and photosynthesis rate were monitored (Figure 5). GCHK1&2 plants irrigated with regular water had significantly lower transpiration rates and stomatal conductance than WT plants irrigated with regular water until flowering, at which point the transpiration rates of both the WT and GCHXK lines increased and became similar (Figure 5). Irrigation with salty water started on Day 41 and LI-COR6800 measurements were taken 10 days later, to allow the plants time to adapt. Irrigation with salty water resulted in significant reductions in the transpiration and stomatal conductance by more than 70% and 50%, respectively of both WT and GCHXK plants. The rate of photosynthesis was also inhibited by more than 50%. However, the rates of transpiration, stomatal conductance and the photosynthesis of line GCHXK2 on Day 59, 18 days after the start of the salt treatment, were slightly but significantly less affected.

**Figure 6:**
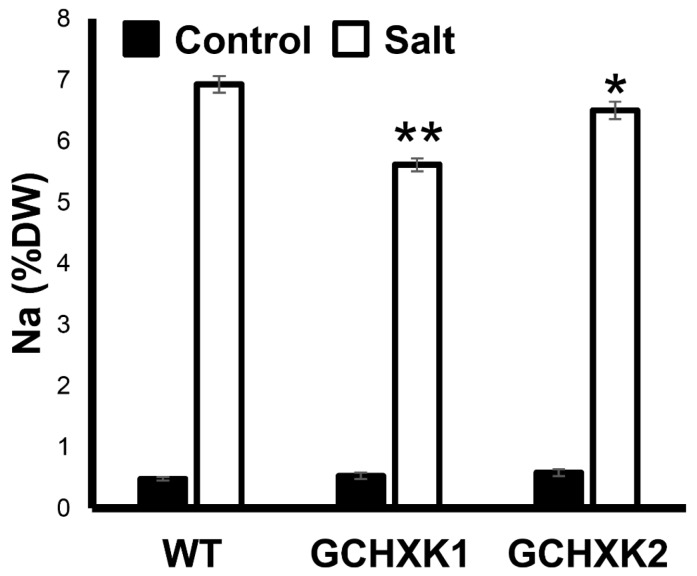
GCHXK plants under salt stress accumulates significantly less Na than WT plants. Na contents of GCHXK and WT plants under saline (white) and control (black) conditions. Data given as means ± SE, *n* = 9. Asterisks denote significant differences relative to the WT (*t*-test, * *p* < 0.05; ** *p* < 0.01).

Measurements of the Na contents of shoots at the end of the experiment revealed that plants irrigated with salty water accumulated about seven times more Na in their dry weight biomass, with GCHXK plants accumulating slightly, but significantly less Na than WT plants (Figure 6). This suggests that less salt was taken up by the GCHXK plants.

**Figure 7:**
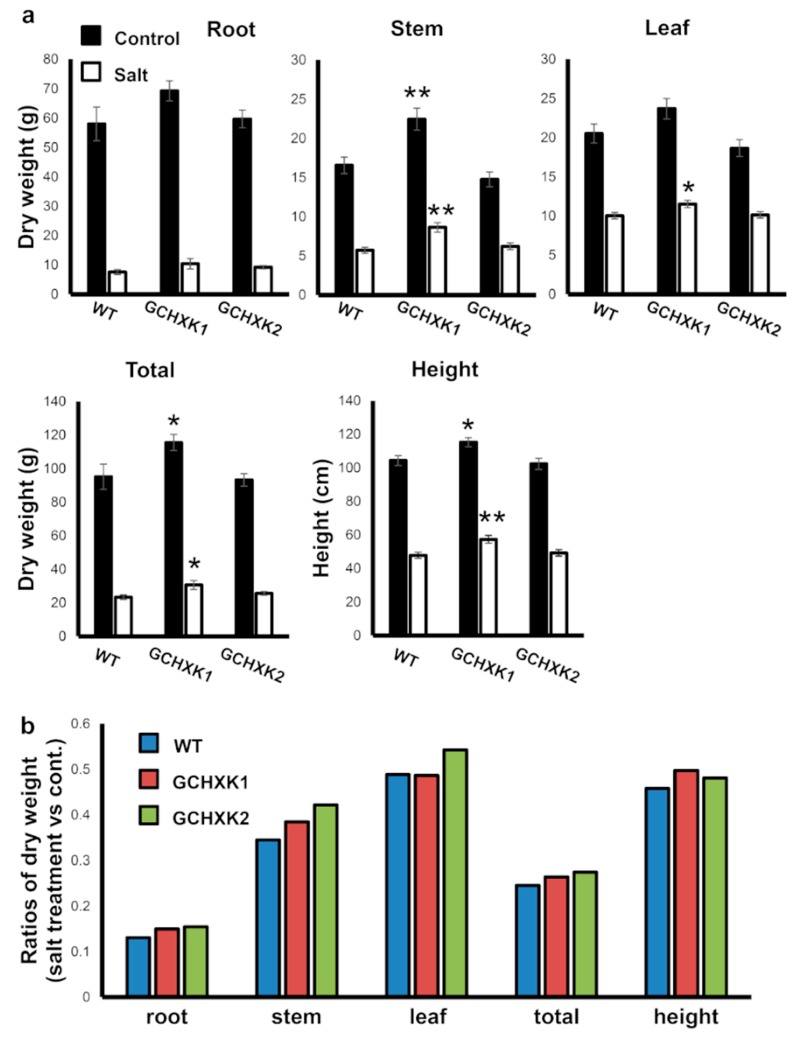
Biomass accumulation of GCHXK plants under saline conditions. GCHXK and WT plants were planted in perlite (an inert growth substance) and allowed to become established and grow for about 90 days. At the end of that period, we started irrigating half of the plants with 150 mM NaCl and continued to irrigate the other half of the plants with regular water. After 20 days of salt treatment, the plants were harvested for dry-weight measurements, *n* = 9. (**a**) Roots, stems, leaves and whole-plant dry weights of control and salt-treated plants and heights of control and salt-treated plants. (**b**) Ratios between the root, stem, leaf and total dry weights and heights of the salt-treated relative to the control plants. Data given as means (± SE). Asterisks denote significant differences relative to the WT (*t*-test, * *p* < 0.05; ** *p* < 0.01).

The biomasses of the salt-treated and control plants were analyzed at the end of the experiment (60 days after planting). Irrigation with salty water significantly inhibited growth, so that root, stem, leaf and total biomass, as well as plant height were reduced by more than 50% in the treated plants (Figure 7). The ratio between the dry weights of salt-treated and regularly irrigated plants of each line are presented in Figure 7b. These ratios demonstrate that roots were most severely affected (85% reduction) by salt irrigation, the stems experienced about a 60% reduction and the leaves exhibited about a 50% reduction, so that the total biomass (roots, stem and leaves) reduction was about 70%. Yet, the values of the biomass ratios and the height ratio of the GCHXK plants were slightly greater than those of the WT plants, perhaps indicating some advantage for GCHXK lines under saline conditions.

## 3. Discussion

The specific expression of *AtHXK1* in guard cells of tobacco plants reduced stomatal conductance, decreased transpiration, and increased WUE by more than 25%, with no negative effects on photosynthesis, plant growth, or development. Similar reductions in stomatal conductance and transpiration rates have been observed in Arabidopsis, tomato and citrus plants [10,24]. Furthermore, the diurnal patterns of transpiration among the various species are very similar (i.e., reduced transpiration primarily in the middle of the photoperiod, when supposedly photosynthesis rates are high, and more sugar is produced and carried by the transpiration stream toward the guard cells (Figure 3b). It is, therefore, likely that sugars stimulate stomatal closure only when the sugar concentration exceeds a certain threshold level, which probably occurs toward midday. Similarly, it has been shown in Arabidopsis that sugar treatments stimulate stomatal closure only when the sugar concentrations exceeds 10 mM [21]. This hypothesis is also supported by the observation that the transpiration rates of the WT and GCHXK plants increased toward flowering time (Figure 5) and that this increase was accompanied by a diminished difference between the transpiration rates of the GCHXK and WT plants. Flowering forms a strong sink for sugars and is known to reduce sugar levels in leaves, which may increase stomatal conductance and transpiration [28,29,30,31]. Thus, the current results support the notion that the sensing of sugar levels within guard cells by HXK coordinates leaf sugar levels with transpiration.

It is generally accepted that reducing stomatal conductance (*g*_s_) should lower the amount of CO_2_ taken up, leading to a lower rate of photosynthesis and decreased biomass production and yield [6,32,33]. However, our GCHXK plants exhibited reduced stomatal conductance and transpiration with no negative effects on their photosynthesis and growth (Figure 1, Figure 2, Figure 3 and Figure 5). Moreover, the GCHXK1 plants exhibited significantly increased stem and total biomass under regular conditions, as compared to the WT plants (Figure 7), indicating that the stomatal behavior of this line improved its physiological properties, allowing greater biomass production, probably through a better balance between water loss and CO_2_ uptake. The unexpected observation of reduced stomatal conductance with no restriction on CO_2_ assimilation may indicate that maximal stomatal conductance is not absolutely necessary for the achievement of high rates of photosynthesis. Alternatively, the stomatal apertures and photosynthesis rates of GCHXK plants might fluctuate less than those of WT plants, thereby compensating for a putative, potentially lower level of CO_2_ stomatal conductance.

### 3.1. GCHXK and Drought

GCHXK appears to have two effects with regard to transpiration: (1) reducing transpiration under well-irrigated conditions (Figure 1, Figure 2 and Figure 3) and (2) attenuating the reduction of transpiration imposed by prolonged drought conditions (Figure 2b and Figure 3b). The continually lower transpiration rate of GCHXK inevitably led to a slower decline in SWC (Figure 3a). In this manner, GCHXK plants were able to delay their exposure to drought conditions and attenuate the reduction in their transpiration. Accordingly, GCHXK plants are capable of making better use of the soil water and are less sensitive to drought stress. This kind of strategy is termed drought avoidance [34] or soil water deficit avoidance [35] and confers the ability to survive longer periods of drought while maintaining rather high levels of productivity. Drought avoidance might be a major advantage in the field, where plants are exposed to episodes of limited water supply. Since total growth depends on the sum of all drought events, the GCHXK plants’ drought avoidance may significantly enhance their overall growth.

### 3.2. Growth-Room Versus Greenhouse Salt Experiments

The reduced transpiration rate of GCHXK plants is accompanied by lower uptake of Na ions, which confers some tolerance to salinity. In the growth-room salt experiment, GCHXK plants accumulated approximately 11% less Na than the WT, and in the greenhouse salt experiment GCHXK1 and GCHXK2 accumulated 19% and 7% less Na, respectively (Figure 4b and Figure 6). The Na contents of the WT and GCHXK plants increased about two-fold upon salt irrigation in the growth room (Figure 4b), but increased about seven-fold upon salt irrigation in the greenhouse, despite the fact that we applied a lower concentration of salt in the greenhouse experiment (150 mM vs. 200 mM in the growth-room experiment). Furthermore, among the plants in the greenhouse, the Na concentration reached about 6% of dry weight; whereas Na accounted for only 0.5% of dry weight in the growth room. It is well-known that growth-room experiments under well-controlled environmental conditions do not necessarily represent real-life greenhouse or field conditions in which the environmental conditions are fluctuating and harsher. For example, we have noticed that the transpiration rate in the greenhouse is usually about four times higher than that observed in our growth room. It is, therefore, not surprising that the salt tolerance of GCHXK in the greenhouse experiment was very marginal and that the application of 150 mM Na had such a significant inhibitory effect on biomass gain (Figure 7). Nevertheless, clearly lower uptake of Na by GCHXK plants, as compared to WT plants, was also evident in the greenhouse experiment and it is likely that more pronounced tolerance might be obtained at lower concentrations of salt. Unlike the marginal tolerance of GCHXK plants to salt observed in the greenhouse, the transpiration rate under regular irrigation (Figure 5) was significantly lower among the GCHXK plants, indicating that the stomatal regulation of GCHXK plants operated well under the fluctuating conditions found in the greenhouse.

With regard to biomass accumulation, no difference was observed between the WT and GCHXK lines when they were irrigated with regular tap water in the growth-room experiment (Figure 4a). Yet, under salty irrigation, GCHXK plants accumulated more than 30% more biomass than the WT plants in the growth-room experiment, in which salt uptake was moderate and was twice as much as with regular irrigation (Figure 4a). In contrast, in the greenhouse experiment, when the plants were irrigated with salty water, significant increases were observed only with GCHXK1 plants. Furthermore, these increases in the biomass of GCHXK1 under salt irrigation could be attributed to the initial increases in GCHXK1 biomass, prior to the exposure to salt. Hence, in the greenhouse experiment in which salt uptake under salty irrigation was about seven times higher than with irrigation with regular water (much higher than in the growth-room experiment), the biomass accumulation of GCHXK plants was only slightly greater than that of the WT plants (Figure 7b). These findings further suggest that the advantage of GCHXK is more pronounced under low salt-stress conditions.

### 3.3. Potential Use of GCHXK

The GCHXK approach for increasing WUE involves a guard cell-specific promoter (*KST1*pro), which drives the expression of Arabidopsis hexokinase1 (*AtHXK1*) specifically in guard cells. Since stomatal closure by sugars appeared early in evolution and is conserved across a diverse group of plants [13], it is likely that the GCHXK approach may have similar positive effects in a wide range of species. Indeed, the GCHXK approach has been proven effective not only in Arabidopsis (*Brassicaceae*) and tomato (*Solanaceae*) [10], but also in citrus (*Rutaceae*) [24]. We, therefore, believe that the GCHXK method could potentially be used in diverse crop species to save water and confer both drought avoidance and tolerance of mild salinity.

## 4. Materials and Methods

### 4.1. Plant Material and Experimental Conditions

*Nicotiana tabacum* cv. Samsun NN plants were used for the experiments in this study. In the greenhouse, the plants were grown in a mixture of 70% tuff and 30% peat (Even Ari, Israel). In the growth room, the plants were grown in soil that contained (w/w) 30% vermiculite, 30% peat, 20% tuff, and 20% perlite (Even Ari, Israel). Plants were grown in a temperature-controlled greenhouse under natural conditions or in a growth room kept at 25°C with a 16 h light/8 h dark photoperiod.

### 4.2. Generation of Transgenic Plants

Transformations were performed using *Agrobacterium tumefaciens* strain EHA105 harboring the pGreen binary vector conferring kanamycin-resistance to plant cells, containing *KSTpro::AtHXK1* (HXK1 locus: *AT4G29130*), as described in [10] and [36,37]. Transgenic plants were identified using a kanamycin-resistance assay and PCR confirmation of the transgene within T_0_ plants. Identification of homozygous plants among T_1_ seedlings was conducted using the TaqMan method [38], with modifications that enabled us to follow the T-DNA copy number in the transgenic plants. Briefly, DNA was extracted from 10 T_1_ plants of each line, the DNA concentration was adjusted to 50 ng/µl in all samples and samples were taken for real-time PCR using primers matching the *nptII* gene present in the T-DNA (Figure S1; Table S1). We suspected that samples with lower Ct contained more T-DNA copies and were homozygous. To confirm homozogosity, seeds were collected from individual plants that were suspected to be homozygous and PCR was used to test 20 seedlings from each individual parent for the presence of the transgene, using primers matching *KSTpro* (forward) and *AtHXK1* (reverse; Table S1).

### 4.3. Whole-Plant Relative Transpiration and Continuous Transpiration-Rate Measurements

Whole-plant transpiration rates were determined using lysimeters, as described in detail in [27]. WT plants and GCHXK transgenic plants were planted in 3.9-L pots and grown under controlled conditions in the greenhouse. Each pot was placed on a temperature-compensated load cell with digital output. Each pot was sealed to prevent evaporation from the surface of the growth medium. A wet vertical wick made of 0.14-m^2^ cotton fibers partially submerged in a 1-L water tank was placed on a similar load cell. Water loss from the wicks provided a reference for the effect of ambient conditions on transpiration. The output of the load cells was monitored every 10 s and the average readings over 3 min were logged in a data-logger for further analysis. Whole-plant transpiration was calculated as a numerical derivative of the load cell output following a data-smoothing process [27]. At the time of the experiment, sunrise was around 5:10 and sunset was around 18:15. For the drought experiment, following a period of normal irrigation with tap water supplemented with liquid fertilizer (4-2-6 Shaphir Nitrate Solution, Gat, Israel) at a concentration of 0.1%, plants were subjected to drought by fully stopping the irrigation for eight days, from Days 11 to 18 (Figure 2 and Figure 3). Volumetric soil water content (SWC) was determined automatically using the algorithm of the lysimeter system, which calculates continuous SWC based on the maximal volumetric water capacity of the soil.

### 4.4. Salt Stress in the Growth Room

To observe the effects of salt on the whole plants, 3-week-old plants that had been germinated in soil and kept in a growth room at 25°C with a 16 h light/8 h dark photoperiod were watered for 1 month with regular tap water or with tap water containing 200 mM NaCl. After 1 month, the plants were harvested and dried at 65°C for 72 h, and the dry weight of each sample was then measured.

### 4.5. Salt Stress–Greenhouse Experiment

To observe the effects of the salt treatment on the whole plant in the greenhouse, 4-week-old plants that had been germinated in soil (in a growth room kept at 25°C with a 16 h light/8 h dark photoperiod) were transferred to the greenhouse, where they were transplanted into 3-L pots that contained perlite. After 41 days in the greenhouse, the plants were watered with tap water containing 150 mM NaCl and fertilizer or with regular tap water and fertilizer as a control for 20 days.

### 4.6. Dry Weight Measurements and Sodium Concentration

Plant samples from the growth-room salt experiment and from the greenhouse salt experiment (all grown in soil) were washed with distilled water to avoid external contamination. The samples were dried at 65 °C for 72 h and the dry weight of each sample was measured. The Na contents of shoots of plants from the growth-room salt experiment were evaluated using four samples composed of three to four shoots (each taken from an individual plant) for each line and treatment. For the greenhouse salt experiment, all of the leaves and the whole stem from each individual plant were collected (*n* = 9). The sampled tissue was ground to a fine powder and 100 mg of the powder were digested with sulfuric acid and peroxide [39]. Na concentration was determined using atomic absorption (AA 800, Perkin Elmer, Norwalk, CT, USA) as described in [40,41].

### 4.7. Gas-Exchange Measurements

Tobacco stomatal conductance (*g*_s_), photosynthesis and transpiration rates were measured on fully developed leaves (Leaf 4-5 from the top) of plants grown in a greenhouse. For the experiment described in Figure 1, the age of the plants was 1.5 months. For the greenhouse salt experiment, the age of the plants is indicated in the figures for each measurement. Measurements were conducted using a LI-COR6800 portable gas-exchange system (LI-COR, Lincoln, NE, USA). All measurements were conducted between 8:30 a.m. and 10:00 a.m. Photosynthesis was induced under optimal light (1000 μmol m^−2^ s^−1^) with 400 μmol mol^−1^ CO_2_ surrounding the leaf (Ca). The amount of blue light was set to 10% photosynthetically active photon flux density to optimize stomatal aperture. The leaf-to-air vapor pressure deficit (VPD) was kept between 0.9 and 1.4 kPa during all measurements. Leaf temperature for all measurements was approximately 25°C (ambient temperature).

### 4.8. RNA Extraction, cDNA Preparation and Quantitative Real-Time PCR

Leaf tissue was harvested from WT and GCHXK plants and total RNA was extracted from that tissue using the LogSpin method [42]. In brief, samples were ground using a Geno/Grinder (SPEX SamplePrep, Metuchen, NJ, USA) and RNA was extracted in 8M guanidine hydrochloride buffer (Duchefa Biochemie) and transferred to tubes containing 96% EtOH (BioLab, Jerusalem, Israel). Then, samples were transferred through a plasmid DNA extraction column (RBC Bioscience, New Taipei City, Taiwan), followed by two washes in 3M Na-acetate (BDH Chemicals, Mumbai, India) and two washes in 75% EtOH, and eluted with DEPC (diethylpyrocarbonate) water (Biological Industries Israel Beit Haemek LTD, Kibbutz Beit Haemek, Israel) that had been preheated to 65°C. The RNA was treated with RQ1-DNase (ProMega, Madison, WI, USA) according to the manufacturer’s instructions, to degrade any residual DNA. For the preparation of cDNA, total RNA (1 μg) was taken for reverse transcription-PCR using qScriptTM cDNA Synthesis Kit (Quanta BioSciences, Gaithersburg, MD, USA) following the manufacturer’s instructions. cDNA samples were diluted 1:5 in double-distilled water. Quantitative real-time PCR reactions were performed using SYBR Green mix (Thermo-Scientific, Waltham, MA, USA) and reactions were run in a RotorGene 6000 cycler (Corbett, Mortlake, NSW, Australia). Following an initial pre-heating step at 95°C for 15 min, there were 40 cycles of amplification each consisting of 10 s at 95°C, 15 s at 55°C, 10 s at 60°C, and for 20 s at 72°C. Results were analyzed using the RotorGene software. The expression levels of *AtHXK1* were normalized using tobacco actin (XM_016658252) as a reference gene and _ΔΔ_Ct to calculate the expression level. (A list of primers and accession numbers is presented in Table S1.)

### 4.9. Statistical Analysis

Statistical analyses were performed using JMP 5.0 software. The Student’s *t*-test was used to generate every *p*-value. Means were considered to be significantly different at *p* < 0.05.

## Supplementary Materials

The following are available online at https://www.mdpi.com/2223-7747/8/12/613/s1, Figure S1: Schematic illustration of the T-DNA construct used to create GCHXK transgenic lines, Table S1: Primers list.

## References

[1] Golldack D., Li C., Mohan H., Probst N. (2014). Tolerance to drought and salt stress in plants: Unraveling the signaling networks. Front. Plant Sci..

[2] Morison J.I., Baker N.R., Mullineaux P.M., Davies W.J. (2008). Improving water use in crop production. Philos. Trans. R. Soc. B Biol. Sci..

[3] Thompson A.J., Andrews J., Mulholland B.J., McKee J.M., Hilton H.W., Horridge J.S., Farquhar G.D., Smeeton R.C., Smillie I.R., Black C.R. (2007). Overproduction of abscisic acid in tomato increases transpiration efficiency and root hydraulic conductivity and influences leaf expansion. Plant Physiol..

[4] Yoo C., Pence H., Hasegawa P., Mickelbart M. (2009). Regulation of transpiration to improve crop water use. Crit. Rev. Plant Sci..

[5] Park S.Y., Peterson F.C., Mosquna A., Yao J., Volkman B.F., Cutler S.R. (2015). Agrochemical control of plant water use using engineered abscisic acid receptors. Nature.

[6] Flexas J. (2016). Genetic improvement of leaf photosynthesis and intrinsic water use efficiency in C_3_ plants: Why so much little success?. Plant Sci..

[7] Antunes W.C., de Menezes Daloso D., Pinheiro D.P., Williams T.C.R., Loureiro M.E. (2017). Guard cell-specific down-regulation of the sucrose transporter SUT1 leads to improved water use efficiency and reveals the interplay between carbohydrate metabolism and K^+^ accumulation in the regulation of stomatal opening. Environ. Exp. Bot..

[8] Taiz L., Zeiger E. (1998). Plant Physiology.

[9] Granot D., Kelly G. (2019). Evolution of guard-cell theories: The story of sugars. Trends Plant Sci..

[10] Kelly G., Moshelion M., David-Schwartz R., Halperin O., Wallach R., Attia Z., Belausov E., Granot D. (2013). Hexokinase mediates stomatal closure. Plant J..

[11] Li Y., Xu S., Gao J., Pan S., Wang G. (2016). Glucose- and mannose-induced stomatal closure is mediated by ROS production, Ca^2+^ and water channel in *Vicia faba*. Physiol. Plant..

[12] Hei S., Liu Z., Huang A., She X. (2017). The regulator of G-protein signalling protein mediates D-glucose-induced stomatal closure via triggering hydrogen peroxide and nitric oxide production in Arabidopsis. Funct. Plant Biol..

[13] Kottapalli J., David-Schwartz R., Khamaisi B., Brandsma D., Lugassi N., Egbaria A., Kelly G., Granot D. (2018). Sucrose-induced stomatal closure is conserved across evolution. PLoS ONE.

[14] Dennis D., Blakeley S., Buchanans B.B., Gruissem W., Jones R.L. (2000). Carbohydrate metabolism. Biochemistry & Molecular Biology of Plants.

[15] Granot D. (2007). Role of tomato hexose kinases. Funct. Plant Biol..

[16] Granot D. (2008). Putting plant hexokinases in their proper place. Phytochemistry.

[17] Outlaw W.H., De Vlieghere-He X. (2001). Transpiration rate. An important factor controlling the sucrose content of the guard cell apoplast of broad bean. Plant Physiol..

[18] Ewert M., Outlaw W., Zhang S., Aghoram K., Riddle K. (2000). Accumulation of an apoplastic solute in the guard-cell wall is sufficient to exert a significant effect on transpiration in *Vicia faba* leaflets. Plant Cell Environ..

[19] Kang Y., Outlaw W.H., Andersen P.C., Fiore G.B. (2007). Guard-cell apoplastic sucrose concentration—A link between leaf photosynthesis and stomatal aperture size in the apoplastic phloem loader *Vicia faba* L.. Plant Cell Environ..

[20] Lu P., Outlaw W.H., Smith B.G., Freed G.A. (1997). A new mechanism for the regulation of stomatal aperture size in intact leaves. Accumulation of mesophyll-derived sucrose in the guard-cell wall of *Vicia faba*. Plant Physiol..

[21] Medeiros D.B., de Souza L.P., Antunes W.C., Araújo W.L., Daloso D.M., Fernie A.R. (2018). Sucrose breakdown within guard cells is a substrate for glycolysis and glutamine biosynthesis during light-induced stomatal opening. Plant J..

[22] Moore B., Zhou L., Rolland F., Hall Q., Cheng W.H., Liu Y.X., Hwang I., Jones T., Sheen J. (2003). Role of the Arabidopsis glucose sensor HXK1 in nutrient, light, and hormonal signaling. Science.

[23] Rolland F., Baena-Gonzalez E., Sheen J. (2006). Sugar sensing and signaling in plants: Conserved and novel mechanisms. Annu. Rev. Plant Biol..

[24] Lugassi N., Kelly G., Fidel L., Yaniv Y., Attia Z., Levi A., Alchanatis V., Moshelion M., Raveh E., Carmi N. (2015). Expression of Arabidopsis hexokinase in citrus guard cells controls stomatal aperture and reduces transpiration. Front. Plant Sci..

[25] Kelly G., Lugassi N., Belausov E., Wolf D., Khamaisi B., Brandsma D., Kottapalli J., Fidel L., Ben-Zvi B., Egbaria A. (2017). The *Solanum tuberosum KST1* partial promoter as a tool for guard cell expression in multiple plant species. J. Exp. Bot..

[26] Gago J., Douthe C., Florez-Sarasa I., Escalona J.M., Galmes J., Fernie A.R., Flexas J., Medrano H. (2014). Opportunities for improving leaf water use efficiency under climate change conditions. Plant Sci..

[27] Halperin O., Gebremedhin A., Wallach R., Moshelion M. (2016). High-throughput physiological phenotyping and screening system for the characterization of plant-environment interactions. Plant J..

[28] Silber A., Israeli Y., Levi M., Keinan A., Chudi G., Golan A., Noy M., Levkovitch I., Narkis K., Naor A. (2013). The roles of fruit sink in the regulation of gas exchange and water uptake: A case study for avocado. Agric. Water Manag..

[29] Fan P.G., Li L.S., Duan W., Li W.D., Li S.H. (2010). Photosynthesis of young apple trees in response to low sink demand under different air temperatures. Tree Physiol..

[30] Goldschmidt E.E., Huber S.C. (1992). Regulation of photosynthesis by end-product accumulation in leaves of plants storing starch, sucrose, and hexose sugars. Plant Physiol..

[31] Iglesias D.J., Lliso I., Tadeo F.R., Talon M. (2002). Regulation of photosynthesis through source: Sink imbalance in citrus is mediated by carbohydrate content in leaves. Physiol. Plant..

[32] Lawson T., Blatt M.R. (2014). Stomatal size, speed, and responsiveness impact on photosynthesis and water use efficiency. Plant Physiol..

[33] Flexas J., Díaz-Espejo A., Conesa M., Coopman R., Douthe C., Gago J., Gallé A., Galmés J., Medrano H., Ribas-Carbo M. (2016). Mesophyll conductance to CO_2_ and Rubisco as targets for improving intrinsic water use efficiency in C_3_ plants. Plant Cell Environ..

[34] Delzon S. (2015). New insight into leaf drought tolerance. Funct. Ecol..

[35] Gilbert M.E., Medina V. (2016). Drought adaptation mechanisms should guide experimental design. Trends Plant Sci..

[36] Horsch R.B., Fry J.E., Hoffmann N.L., Eichholtz D., Rogers S.G., Fraley R.T. (1985). A simple and general method for transferring genes into plants. Science.

[37] Gallois P., Marinho P., Jones H. (1995). Leaf disk transformation using *Agrobacterium tumefaciens*-expression of heterologous genes in tobacco. Plant Gene Transfer and Expression Protocols.

[38] German M.A., Kandel-Kfir M., Swartzberg D., Matsevitz T., Granot D. (2003). A rapid method for the analysis of zygosity in transgenic plants. Plant Sci..

[39] Snell F.D., Snell C.T., Snell C.A. (1959). Colorimetric methods of analysis. Soil Sci..

[40] Yasuor H., Tamir G., Stein A., Cohen S., Bar-Tal A., Ben-Gal A., Yermiyahu U. (2017). Does water salinity affect pepper plant response to nitrogen fertigation?. Agric. Water Manag..

[41] Levy Y., Raveh E., Lifshitz J. The effect of rootstock and nutrition on the response of grapefruit trees to salinity. Proceedings of the International Society of Citriculture IX Congress.

[42] Yaffe H., Buxdorf K., Shapira I., Ein-Gedi S., Moyal-Ben Zvi M., Fridman E., Moshelion M., Levy M. (2012). LogSpin: A simple, economical and fast method for RNA isolation from infected or healthy plants and other eukaryotic tissues. BMC Res. Notes.

