# Supplementary material for: Expression of Arabidopsis Hexokinase in Tobacco Guard Cells Increases Water-Use Efficiency and Confers Tolerance to Drought and Salt Stress
Source: Plants (Basel). 2019 Dec 16;8(12):613. doi: 10.3390/plants8120613 (PMC6963886; doi:10.3390/plants8120613)
Supplement: Supplementary file 1 [file plants-08-00613-s001.zip › Figure S1.docx]

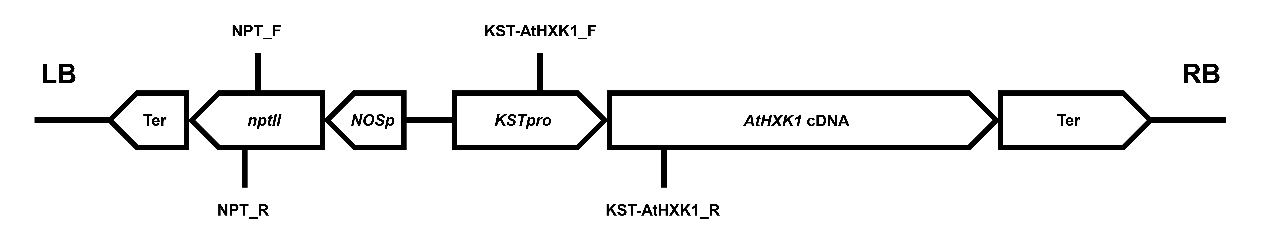


**Figure S1. Schematic illustration of the T-DNA construct used to create GCHXK transgenic lines.** The T-DNA contains kanamycin-resistance gene (*nptII*) under the *NOS* promoter (*NOSp*) and terminator (Ter), and *AtHXK1* cDNA under the *KST1* partial promoter (*KSTpro*) and a terminator. The location of the forward (F) and reverse (R) primers that were used for TaqMan and PCR analyses are indicated above and below the scheme. LB, left border; RB, right border.
