# Supplementary material for: Expression of Arabidopsis Hexokinase in Tobacco Guard Cells Increases Water-Use Efficiency and Confers Tolerance to Drought and Salt Stress
Source: Plants (Basel). 2019 Dec 16;8(12):613. doi: 10.3390/plants8120613 (PMC6963886; doi:10.3390/plants8120613)
Supplement: Supplementary file 1 [file plants-08-00613-s001.zip › Table S1.pdf]

Table. S1

**Primers list**			
Gene	Forward primer (5’-3’)	Reverse primer (5’-3’)	Accession
AtHXK1	AAACCTACCCAAAGAGCGCC	TGACGCCTTAGAACTTGGCT	AT4G29130
ACTIN	CCTGAGGTCCTTTTCCAACCA	GATTCCGGCAGCTTCCATT	XM_016658252
NPT	CCGGCTACCTGCCCATTC	CGACAAGACCGGCTTCCA	DQ449894
KST - AtHXK1	AGGCAAGTAGCAATGTCACG	CAGTTTCGAGATCGGAGTCG	
